# Morphometry around Cochleostomy Site to Aid Safe Cochlear Implantation

**DOI:** 10.22038/IJORL.2023.65579.3249

**Published:** 2023-05

**Authors:** Yellur Kavitha, Upendra Kumar Joish

**Affiliations:** 1 *De* *partment of ENT, * *SDM College of Medical Sciences and Hospital, SDM University, Dharwad,* * Karnataka, India.*; 2 *Department of Radiology, SDM College of Medical Sciences and Hospital, SDM University, Dharwad, Karnataka, India.*

**Keywords:** Basal turn diameter, Cochlear implantation, Cochlear ossification, Round window to carotid canal distance, Promontory thickness

## Abstract

**Introduction:**

In morphologically normal-appearing inner ears, measurements of the distance between the round window and carotid canal (RCD), the maximum diameter of the basal turn of the cochlea next to the round window (BD), and the thickness of the promontory (PT) just lateral to the basal turn may be used as guide for safe cochleostomy and implant placement.

**Materials and Methods:**

From January to March 2022, a cross-sectional observational study was carried out in a tertiary care hospital. The round window to carotid canal distance (RCD), the largest diameter of the cochlea's basal turn next to the round window (BD), and the thickness of the promontory immediately lateral to the basal turn (PT) were measured using CT temporal bone images of 150 persons without cochlear abnormalities. The values obtained were compared using Paired T-test for significance of difference between both genders and sides.

**Results:**

A total of 150 participants—75 men and 75 women—with a mean age of 37.5 years were enrolled in the study. With a range of 7.18 mm to 10.52 mm, the mean RCD was 8.84 mm (SD 0.8 mm). The mean BD was 2.27 mm (SD 0.4 mm), while the mean PT was 1.15 mm (SD 0. mm). The values obtained did not differ significantly in both the genders and the right and left sides (p = 0.37 and 0.24, respectively).

**Conclusion:**

The present study has defined and calculated pertinent measures at cochleostomy site that will aid safe electrode insertion and prevent misplacement.

## Introduction

Cochlear implants have proved to be boon to patients with sensorineural hearing loss (SNHL) occurring due to various causes ranging from congenital SNHL to presbycusis. They have a profound effect on these people's social lives and self-confidence in addition to improving their hearing and communication skills. The delicate, microscopic procedure of cochlear implantation relies on exact anatomical landmarks in the middle and inner ears. Preoperative cross-sectional imaging, such as CT and MRI scans, obviously gives the surgeon crucial information about those anatomical landmarks, variations, and anomalies, if any. Though uncommon, electrode misplacement (incidence 0.2 to 2.1%) is a significant problem. The sites of misplacement mentioned include the vestibule, carotid canal, Eustachian tube, internal auditory canal, semicircular canals, and petrous apex; these sites require re-implantation or repeat surgery as a result of implant failure. Carotid canal insertion can be potentially life threatening (1-4). 

Ossification of membranous cochlea due to otosclerosis, middle and inner ear infections, meningitis and idiopathic forms and congenital inner ear anomalies like partition anomalies, complicate the anatomy and perceived depth of insertion of electrodes. For the implant to work effectively in ossified cochleae, electrodes must be put into the relatively un-ossified parts of the cochlea (5–6). 

Mastoidectomy and posterior tympanotomy are the two surgical procedures most frequently used for cochlear implantation (7-8). 

Based on this surgical strategy, analysis of multiple CT images was performed and three measurement parameters were identified which could potentially help decrease the likelihood of electrode misplacement, especially into the carotid canal. 

These parameters were chosen after wider consultation and considering the criteria of ease of measurement on preoperative CT scans and their real-time applicability during surgery. The three parameters were distance between round window and carotid canal (RCD), maximum diameter of basal turn of cochlea adjacent to round window (BD) and thickness of promontory (PT) just lateral to basal turn adjacent to round window. This study was done to estimate typical values of these parameters in inner ears that seemed morphologically normal.

## Materials and Methods

A prospective, cross-sectional study was undertaken in a tertiary care centre involving 150 adult individuals (75 males and 75 females) who underwent head CT scans for any indication between December 2021 and February 2022 after taking institutional ethics committee clearance and informed consent of individuals. Sample size was calculated using precision-based sample size calculation formula. 

The Somatom Emotion 16-slice CT scanner was used for the CT scans. (Siemens, Erlangen, Germany). The source images were used to reconstruct 1mm thick, high-resolution temporal bone window images in the axial plane. The image datasets were anonymised before being viewed on Osirix application by a Radiologist with 9 years of experience in head and neck imaging. The study excluded any individuals in whom middle or inner ear abnormalities were found on CT scan.

The following parameters were measured bilaterally using straight line tool in each case.

**Fig 1:**
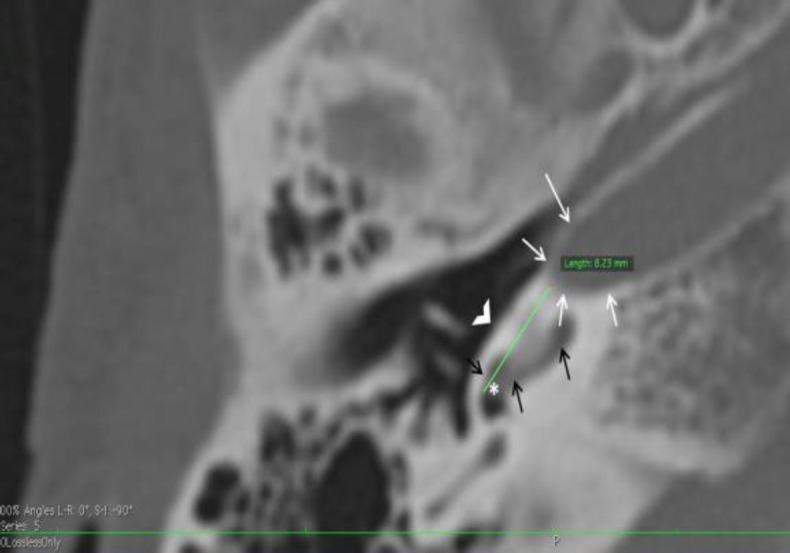
Axial CT image of temporal bone illustrating the method of measuring round window to carotid canal distance (RCD) which is represented by the green straight line. Note that the measurement is the shortest distance from anterior margin of round window (asterisk indicates round window) to the carotid canal (white arrows) and parallel to the promontory (open white arrowhead). The black arrows point at the Basal turn of cochlea

**Fig 2:**
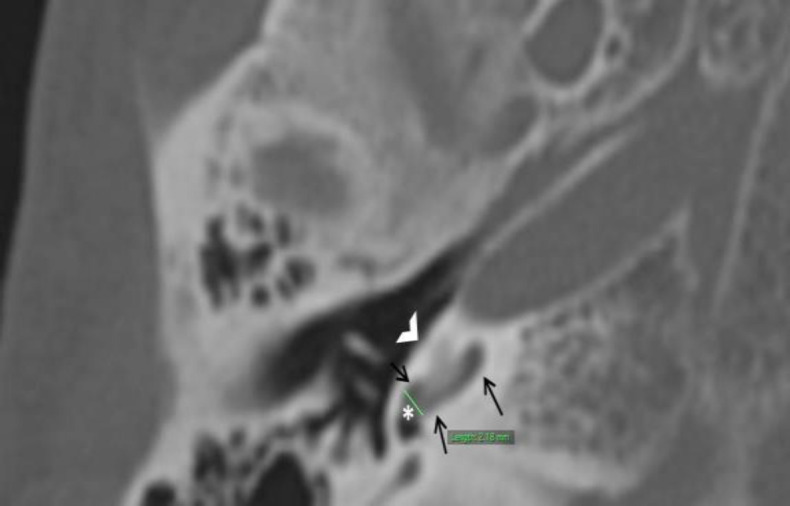
Axial CT image of temporal bone demonstrating method used to measure diameter of basal turn of cochlea (BD) as depicted by straight green line. Note that the measurement is done adjacent to the round window (asterisk). Basal turn of cochlea is denoted by black arrows while white open arrow heads indicate promontory

**Fig 3:**
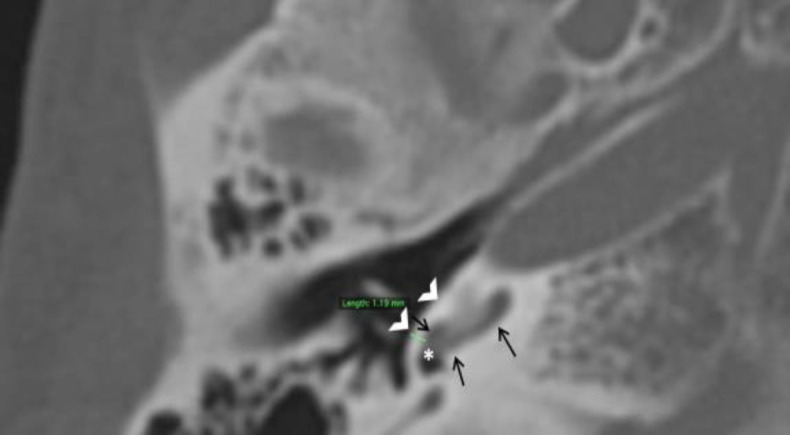
Axial CT image of temporal bone demonstrating method used to measure thickness of promontory (PT) as depicted by straight green line. Note that the measurement corresponds to lateral bony wall of basal turn of cochlea (black arrows) adjacent to the round window (asterisk). Promontory is indicated by white open arrow heads

- Round window to carotid canal distance (RCD) is the shortest distance between the anterior end of round window and the Carotid canal on axial CT image measured parallel to promontory in that image as shown in figure 1.

- Maximum diameter of basal turn of Cochlea adjacent to round window (BD) as depicted in figure 2.

- Thickness of promontory/lateral bony wall of basal turn of cochlea adjacent to round window (PT) as illustrated in figure 3.

The data was tabulated using Statistical product and service solutions (SPSS, version 22). Mean values and standard deviations of the values were calculated separately for each side and males and females. Significance of any differences in values between the two genders and sides was determined using Paired T test.

## Results

The study involved 150 individuals in total, 75 of whom were men and 75 of whom were women. The participants' average age was 37 years and 6 months. Participants obtained head CT scans for a variety of reasons, including trauma (69), headaches (42), giddiness (19), suspected stroke (15), and others (5).Overall, the mean RCD was 8.84 mm (SD 0.8 mm) with a range of 7.18 mm to 10.52 mm. Mean BD was 2.27 mm (SD 0.4 mm) and PT was 1.15 mm (SD 0.2 mm). There was no significant difference between right and left sides (p = 0.24) and between both the genders (p = 0.37). 

## Discussion

Estimation of basic and easy to perform morphometry of structures around the cochleostomy site can prove handy for a cochlear implant surgeon while operating a case with cochlear abnormality in order to avoid complicationsAdditionally, the inferior segment of the cochlea's basal turn and the round window area are frequent locations for ossification; as a result, a preoperative evaluation of these structures is essential for the surgeon to plan the depth of bone drilling necessary for a secure and effective cochlear implantation (9). The three measurements defined and estimated in this study are close to the cochleostomy site and provide useful information about amount of cochlear ossification if any (PT and BD), the available cochlear luminal diameter (BD), the depth of electrode insertion (PT) and the distance of carotid canal from cochleostomy site (RCD).

***RCD***

Distance from round window niche to carotid canal (RCD) was found to be 8.03±1.55mm by Singla et al (10) and 8.08±1.55 mm by Wysocki et al (11) in cadavers and 9.4 mm by Yilmazer et al on CT images (12). These measurements are comparable to the mean RCD recorded in the current study (8.84 mm). However, Yilmazer et al measured RCD from infero-medial part of round window whereas in the present study, measurement was done from antero-inferior margin of round window which is the commonest site for cochleostomy. Jain et al (13) who studied photographs of dissected temporal bone specimens, found the range of RCD between 2.79 mm and 5.34 mm, which is much smaller in comparison with the present study. Additionally, compared to the research described above, the sample size of the current study is bigger. In order to prevent electrode implantation into the carotid canal, RCD is a crucial parameter. The risk increases with proximity. The depth of bone drilling at the site of the cochleostomy should not be greater than 5–6 mm, as seen from the aforementioned numbers.

***BD***

Diameter of basal turn of cochlea measured in the present study closely corresponds to the inner diameter of basal turn of cochlea at 0 degrees as measured by Singla et al (14). Singla et al measured it on photographs of dissected temporal bone specimens and reported that it was 1.98 mm, but in the current work it was measured on CT images and found to be 2.27 mm. In earlier studies, maximum diameter of basal turn of cochlea was found to be 1.75 mm (15) and 1.81 mm (16), which were obtained on virtually uncoiled cochlear images on MRI; hence depicting membranous cochlea. BD represents available luminal width for implant insertion. A decrease in the BD signals aberrant ossification and alerts the surgeon as a result since the basal turn is more susceptible to ossification.

***PT***

There isn't much research on this measurement. A closely corresponding measurement by Laurikainen et al found it to be 1.49mm, whereas in the present study it was 1.15mm (17). The difference in values could be due to different methodologies employed. The diverse approaches used could be the cause of the values' discrepancy. Unlike the current work, which used CT scans, Laurikainen et al. quantified it on micro-projected microscopic pictures of dissected specimens. PT suggests minimum depth of bone drilling required to access basal turn of cochlea. Several other measurements have been described in relation to cochleostomy like distances from facial recess and round window to IAC, jugular bulb etc (10,12-13). However, after consultation, majority of surgeons believed that distances from round window would be a better per-operative reference. In the present study, focus was on extent of bone drilling required and avoidance of carotid insertion and only dimensions relevant to them were selected. It is suggested that additional research be conducted to apply these criteria to aberrant and ossified cochleas and discover the effects of their values on surgical simplicity and success.

## Conclusion

Pertinent measurements around the cochleostomy site have been defined and estimated in the present study, which will aid in safe electrode insertion and prevent misplacement. These parameters are suggested to be incorporated in the pre-operative work up for cochlear implantation.
